# Evaluation of a health system intervention to improve virological management in an antiretroviral programme at a municipal clinic in central Durban

**DOI:** 10.4102/sajhivmed.v20i1.985

**Published:** 2019-09-26

**Authors:** Christie M. Cloete, Jane Hampton, Terusha Chetty, Thando Ngomane, Elizabeth Spooner, Linda M.G. Zako, Shabashini Reddy, Tarylee Reddy, Nozipho Luthuli, Hope Ngobese, Gita Ramjee, Anna Coutsoudis, Photini Kiepiela

**Affiliations:** 1 HIV Prevention Research Unit, South African Medical Research Council, Durban, South Africa; 2 eThekwini Health Unit, eThekwini Municipality, Durban, South Africa; 3 School of Clinical Medicine, University of KwaZulu-Natal, Durban, South Africa; 4 Department of Biostatistics, South African Medical Research Council, Durban, South Africa

**Keywords:** HIV-1 infected, antiretroviral care or management, health strengthening systems interventions, virological suppression, retention in care

## Abstract

**Background:**

With the largest antiretroviral therapy (ART) programme globally, demand for effective HIV management is increasing in South Africa. While viral load (VL) testing is conducted, VL follow-up and management are sub-optimal.

**Objectives:**

The objective of this study was to address gaps in the VL cascade to improve VL testing and management.

**Methods:**

Antiretroviral therapy records were sampled for an in-depth review. The study team then reviewed individual records, focusing on ART management, virological suppression and retention. Multifaceted interventions focused on virological control, including a clinical summary chart for ART care; streamlining laboratory results receipt and management; monitoring VL suppression, flagging virological failure and missed visits for follow-up; down-referral of stable patients eligible for the chronic club system; and training of personnel and patients.

**Results:**

Pre-intervention, 78% (94/120) of eligible patients had VL tests, versus 92% (145/158) post-intervention (*p* = 0.0009). Pre-intervention, 59% (71/120) of patients accessed their VL results, versus 86% (136/158) post-intervention (*p* < 0.0001). Post-intervention, 73% (19/26) of patients eligible for ART change were appropriately managed, versus 11% (4/36) pre-intervention (*p* < 0.0001). Only 27% had no regimen changes (7/26) post-intervention, versus 81% (29/36) pre-intervention (*p* < 0.0001).

**Conclusion:**

Service delivery was streamlined to facilitate HIV services by focusing on VL test monitoring, protocol training and accessibility of results, thereby improving clinical management.

## Introduction

South Africa has the largest antiretroviral therapy (ART) programme in the world with over 4.1 million adults on treatment by the end of 2017.^1^ Despite expanded ART access, incident HIV infections are still substantial with approximately 270 000 individuals infected with HIV in 2017.^1^ In 2014, UNAIDS launched the 90–90–90 targets to progress towards elimination of HIV by 2020.^2^ The last ‘90’ of the UNAIDS strategy is to ensure that 90% of individuals receiving ART have achieved virologic suppression.^2^

Plasma viral load (VL) is the main driver of new HIV infections.^3^ HIV replication is interrupted by ART, thereby reducing VL, decreasing transmission risk and improving morbidity and survival.^4^ Post-initiation, VL is indicative of ART response.^5^ In practical terms, to achieve the UNAIDS 90–90–90 goals towards HIV elimination, 73% of people living with HIV would need to be virologically suppressed to reduce the reproductive rate of infection to below 1.^2^

In a study of South Africa’s ART ‘treatment cascade’ using data from the national health laboratory service (NHLS), almost half of all South Africans infected with HIV were not linked to care.^6^ The authors were not able to report the proportion of *diagnosed* people who were not linked to care. However, among those linked to care and on treatment, only 73.7% had suppressed VLs. The overall proportion of viral suppression among South Africans living with HIV was 23.8%.^6^ Part of the challenge of achieving virologic suppression may be the complexity of VL monitoring – from attaining patient blood samples, transport of laboratory specimens, documenting results in patients’ files and appropriate management of virological outcomes.^7^

Moreover, maintaining viral suppression may be particularly challenging with psychosocial, cultural and economic obstacles to adherence, including efficient systems for laboratory monitoring and adequate, timely drug supply.^8^ Given the multifactorial barriers, maintaining drug adherence may necessitate a hierarchical approach with patients at risk triaged for additional clinical and psychosocial support.^8^

The primary objective of this health systems intervention was to address gaps in the VL cascade in a real-world setting to improve VL testing and management. In this article, we present the evaluation of results for this health system intervention.

## Research methods and design

### Study design

The study team conducted a health system intervention to improve the quality of HIV services, focusing on virological management.

### Setting

The eThekwini district of KwaZulu-Natal (KZN) had an HIV prevalence of 11.4% in 2016^9^ and one of the highest antenatal HIV prevalence rates in the country at 41.1% in 2013.^10^ The HIV programme implemented at the Lancers Road Primary Healthcare (PHC) clinic is managed by the eThekwini Health Unit, situated near a busy taxi rank in Durban. This PHC offered a unique opportunity to determine if a quality improvement intervention could improve virological management in a setting servicing a highly mobile population. It offers standard PHC services: antenatal care, well-baby clinic and/or immunisations, HIV counselling and testing, ART provision, tuberculosis (TB) screening and treatment and other chronic care services to patients from the greater functional region within 100 km radius.

#### Routine HIV management

Approximately 60–70 HIV-positive patients were managed daily at the PHC by a single nurse clinician with weekly medical officer support. During the study period from 2011 to 2015, the South African Department of Health (DOH) CD4^+^ threshold for ART initiation was revised from 200 cells/mm^3^ in 2013^11^ to < 500 cells/mm^3^ in 2015.^12^

Once initiated on ART, patients were followed up monthly by nurses who were responsible for clinical and laboratory monitoring, dispensing of medications, re-enforcing adherence messages and providing psychosocial support. Consultations focused on ART initiation and dispensing, with little time for ensuring the accessing and management of VL results, or for comprehensive clinical care such as management of communicable and non-communicable diseases. In addition, there were few opportunities for doctors to mentor nurses. The guideline also recommended down-referral of stable patients who were suppressed virologically to community-based ‘chronic clubs’ for ongoing care.

The current South African guideline recommends VL testing following ART initiation at 6 months, 12 months and thereafter annually.^12^ For patients with VL < 400 copies/mL, VL monitoring may be conducted annually, depending on the ART duration and routine adherence support. If the VL > 50 copies/mL, adherence support should be intensified. Patients with VL 400 copies/mL – 1000 copies/mL should have their adherence carefully assessed and a repeat VL test within 6 months. Patients with VL > 1000 copies/mL should have their adherence assessed, intensive adherence support and a repeat VL test in 2 months; if the VL remains > 1000 copies/mL, the patient should be switched to a second-line ART regimen. If the repeat VL is < 1000 copies/mL, the VL should be repeated in 6 months and reassessed for further management.

### Study population and sampling strategy

All patients (*n* = 1538) enrolled in the ART programme at the PHC facility from 01 September 2011 to 31 March 2014 were included in pre-intervention reviews. Furthermore, the authors systematically sampled 13% of all patients initiated on ART between 01 September 2011 and 31 March 2014 for in-depth file reviews.

Every 10th file was selected for review and recording data captured until end of March 2014. In addition, files of patients commenced on ART in the 3 months following the initial file review period, that is, 01 April 2014–30 June 2014, were reviewed post-intervention.

### Health systems assessment

Our research involved file reviews at several time points: (1) pre-intervention review conducted from 01 April to 30 June 2014 of patients enrolled in the ART programme from 01 September 2011 to 31 March 2014; (2) intervention (01 April 2014 – 31 March 2015) and (3) post-intervention assessment (May 2015 to 31 December 2015) (Table 1).

**TABLE 1:** Pre-intervention, intervention and post-intervention description of activities conducted at the Lancers Road PHC from 01 September 2011 to 31 December 2015.

Study phase	Activity	Description of activities
**Pre-intervention** Conducted from 01 April 2014 to 30 June 2014 of patients enrolled in the ART programme from 01 September 2011 to 31 March 2014	In-depth file review (IDR) (data not shown (*n* = 206)	Assessed baseline clinical management of HIV-positive patients in ART services Evaluated demographics, ART initiation, TB treatment, pregnancy, co-morbidity, psychosocial evaluations, visit data and laboratory results
	VL and retention in care review (VLRIC) (*n* = 1339)	The study team reviewed VL results management and whether clients were retained in care The NHLS computerised system was used to locate VL results not located in client files
	VL management review (VLMR) (*n* = 68)	Assessed all patients with elevated VL from the VL and retention in care review Reviewed clinical management to determine guideline fidelity: follow-up VL testing; adherence counselling; regimen change and time frames for these actions
	Insertion of clinical summary charts (*n* = 1339)	Research clinicians inserted clinical summary sheets in every patient file, management assessed Patients requiring additional intervention were referred to nurse clinicians or sessional medical officers
**Intervention** 01 April 2014–30 April 2015	Training sessions	Clinic staff were offered guideline-based training, including high VL management, CD4+ testing, pregnancy and postpartum ART management, prophylaxis for opportunistic infections, TB diagnosis and management, individual client care and preparation for ‘chronic clubs’, and completion of clinical summary charts Patient education focused on chronic clubs and viral suppression Feedback was given to clinicians managing a client, where appropriate
	Clinical summary chart insertion	Nurses introduced clinical summary charts for all other new clients ART regimen and duration, due date for blood tests, laboratory results and interventions required were summarised High VLs were flagged for intervention, missing results followed up and plans for stable clients to collect medication without a clinical visit
	Improved laboratory systems	Daily filing of thermal printer results sent through the ticker tape system Printing of missing laboratory results from the NHLS website Filing of the hard copies of the laboratory results in the client file for clinical management at the appointment date
	VL management system	High VL results flagged for immediate management Clients without NHLS blood results were noted to require a VL test at the next visit Clients with raised VL results, which were not adequately managed, were called for an appointment with the clinic doctor within a week Unsuppressed VL files were kept in a marked box and not returned to the general filing system until the client had an undetectable VL result
	Follow-up system for missed appointments and lost to follow-up	Patients with missed appointment dates were flagged for follow-up with the help of community health workers
	Chronic club care	Down-referral system to transition stable clients for collection of medication (not requiring clinical consultation) occurred within the clinic, in preparation for down-referral to a community site Basic protocol formulated for patient eligibility, an updated appointment card designed and an educational message for relevant research staff prepared to create awareness in patients Patients eligible for chronic clubs were given dates for medication collection or consultation; using a diary system those with missing appointments could be identified and contacted to improve retention in care
**Post-intervention** May 2015—31 December 2015	Improvement in VL testing and access to VL results (*n* = 170)	Available files from the *pre-intervention in-depth review* were compared to VL testing conducted post-intervention Assessed improvement in VL testing, including VLs conducted and managed
	VL management follow-up (*n* = 68)	Re-assessed the follow-up management of clients identified with a high VL during the *VL and retention in care review (pre-intervention)* between 10 January 2015 and 30 June 2015
	Clinical summary chart review (*n* = 280)	Study team reviewed the uptake of clinical summary sheets on new clients enrolled in the ART programme Research clinicians were not routinely involved in the insertion and completion of the clinical summary charts
	Down-referral system (chronic clubs)	Eligibility for chronic clubs: patients on ART > 12 months; latest VL result undetectable/low detectable limit; not currently pregnant or breastfeeding; no recent change in regimen and clinically well

#### Pre-intervention (Figure 1)

The study team assessed the baseline operational issues of the clinic to guide our interventions and focus. The review was conducted as follows: (1) In-depth file review (IDR); (2) VL and retention in care review (VLRIC); (3) VL management review (VLMR).

**FIGURE 1:**
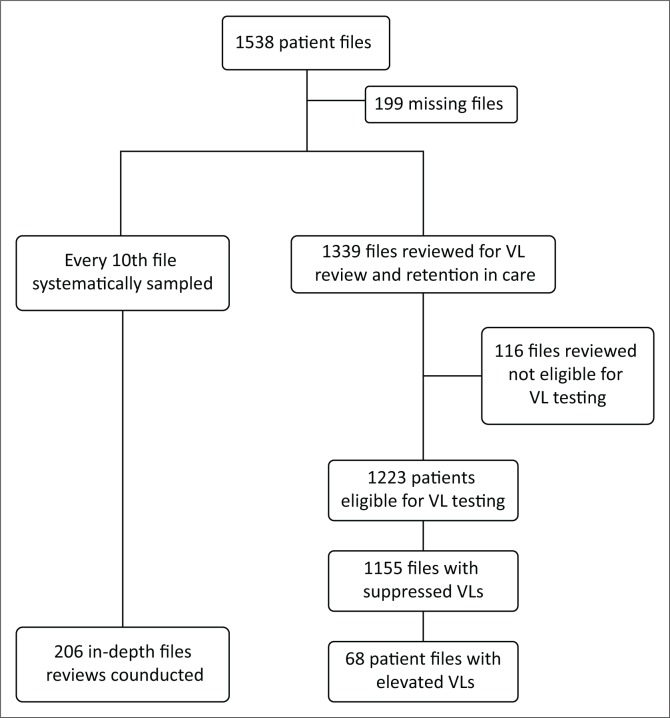
Flow diagram for the health systems intervention conducted at the Lancers PHC from 01 September 2011 to 31 March 2014.

#### Intervention period

**FIGURE 2:**
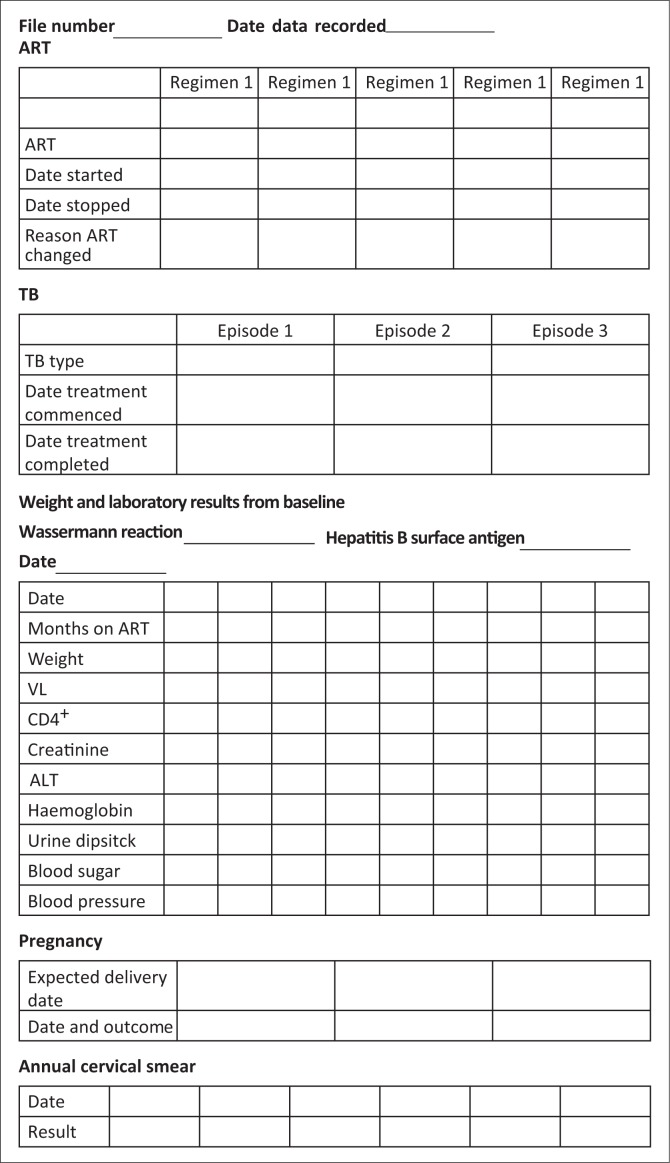
Clinical summary chart.

Based on the in-depth review, the following combination of interventions was implemented: (1) training sessions; (2) clinical summary chart insertion (Figure 2); (3) improved laboratory systems; (4) VL management system; (5) follow-up system for missed appointments and lost to follow-up and (6) chronic club care.

#### Post-intervention assessment

The effectiveness of our combination of interventions at the clinic was measured as follows: (1) improvement in access to VL results, whether performed and included in the client file; (2) VL management follow-up (reassessed clients found with high VL during the VLRIC); (3) clinical summary chart usage review (uptake of clinical summary sheet on new clients enrolled in the ART programme) and (4) down-referral system (chronic clubs).

### Data collection

All pre- and post-intervention data extracted from the patient files were recorded on appropriate questionnaires. The data were entered into a specific database developed by research clinicians. The database was secured using password-protected access systems. All data were de-identified to maintain patient confidentiality; all specific laboratory specimens, reports, study data collection, and administrative forms and folders were identified using the clinic patient number.

### Data analysis

Continuous variables, such as age, were summarised as means with standard deviations, or medians and interquartile ranges, where appropriate. *T*-tests or Wilcoxon rank-sum tests were used to determine whether the distribution of the outcome significantly differed between groups. Categorical variables were analysed using frequency tables, and the chi-square or Fisher’s exact test to test the relationship between two categorical variables.

### Ethical considerations

Permission was granted by the eThekwini Health Research Committee as part of the Memorandum of Understanding (MOU) between the eThekwini Health Unit and the Medical Research Council (MRC) HIV Prevention Research Unit (27 May 2013) for an operational implementation programme to conduct a health systems intervention at the PHC. The eThekwini Health Unit has a MoU with the Department of Health for the use of routine data for research purposes at municipal facilities. Informed consent and study-related documents were not required for the operational implementation programme as no patient contact was involved.

## Results

### Pre-intervention

#### In-depth review

The cohort was predominantly female (77%; 159/206). Median age of females was 32 years and males 36.5 years. Approximately 26% of patients (54/206) were already on ART when transferred into the PHC. Of those initiated at Lancers Road, 64% (97/152) started care with CD4^+^ counts < 200 cells/mm^3^. The majority of patients on ART (76%; 156/206) were on the single-dose regimen of tenofovir, emtricitabine and efavirenz (TDF–FTC–EFV).

#### Viral load and retention in care review (Figure 1)

Viral load testing (test data and recorded results) and retention status were available for 87.1% (1339/1538) of patients. Seventy-nine per cent of patients were retained in care (1058/1339), 15% lost to follow-up (201/1339) and 6% were transferred out (80/1339). There were 116 patients (8.7%) not eligible for VL testing, leaving 1223 patients eligible for VL testing.

Overall, there were 997 VL test results in 1223 eligible patients; 82% of results were in the patients’ files and 18% on the NHLS website. In these 997 patients with VL test results, 73% were suppressed; 3% were 400 copies/mL – 1000 copies/mL; 5% were > 1000 copies/mL; 1% were not processed and 18% were not adequately monitored according to standard guidelines.

One hundred and seventy patient files from the in-depth review cohort (*n* = 206) were used to compare access to VL results pre- and post-intervention. Of the 170 patients with files reviewed, 70.6% (120/170) were eligible for VL testing pre-intervention, while 158/170 were eligible post-intervention. The remaining 50 and 12 patients, respectively, had not yet reached the VL testing period of 6 months and did not have VL tests.

Pre-intervention, 78% (94/120) had VL tests conducted, 76% (71/94) of VL tests were filed and 59% (71/120) had access to their results (Table 2).

**TABLE 2:** Pre- and post-intervention results of health systems evaluation.

Health systems evaluation	Pre-intervention		Post-intervention		Chi-square (*p*)
	%	*n*	%	*n*	
**In-depth review**					
VL tests done	78	94/120	92	145/158	0.0009
Results filed	76	71/94	94	136/145	-
Access to VL results	59	71/120	86	136/158	< 0.0001
**VL management review**					
Results filed	85	58/68	-	-	-
Results acknowledged	43	29/68	-	-	-
Repeat VL	30	21/68	-	-	-
<3 months	3	2/68	-	-	-
3–6 months	6	4/68	-	-	-
>6 months	22	15/68	-	-	-
Eligible for regimen change	53	36/68	38	26/68	
No regimen change	81	29/36	27	7/26	< 0.0001
Any regimen change	19	7/36	-	-	-
Appropriate regimen change	11	4/36	73	19/26	< 0.0001
Inappropriate regimen change	8	3/36	-	-	-
**Clinical summary sheet**					
Documentation	-	-	-	-	-
Filed	-	-	85	224/262	-
Inserted by clinic staff	-	-	92	207/224	-
Inserted by research staff	-	-	7	15/224	-
Assessed unknown	-	-	1	2/224	-
Not filed	-	-	14	36/262	-
Assessed N/A	-	-	1	2/262	-
**VL documentation in the previous year**					
VL recorded	-	-	82	184/224	-
VL not recorded	-	-	11	24/224	-
VL assessed not applicable	-	-	7	16/224	-
VL result in file but not recorded	-	-	6	14/224	-
VL result available (recorded or not)	-	-	88	198/224	-
**Clinical management**					
ART regimen documented	-	-	93	209/224	-
ART regimen documented up-to-date	-	-	98	205/209	-
Undocumented regimen change	-	-	2	4/209	-

Possible reasons for failure to receive a laboratory result were as follows: blood tests not requested nor performed; transport logistics; rejection of blood specimens and misplacing of the printed laboratory result form at the clinic.

#### Viral load management review

Pre-intervention, in the 68 patients with raised VLs, it was noted that only 30% had a repeat VL. Repeat VLs were seldom done timeously in accordance with DOH protocol with 22% conducted > 6 months (Table 2).

Of the 68 patients, 53% (36/68) had virologic failure and were eligible for a regimen change (in care with at least two prior raised VL results). Pre-intervention, of the 36 who were eligible for regimen change, 81% (29/36) had no regimen change when needed, 8% (3/36) had an inappropriate regimen change and 11% (4/36) had appropriate regimen change according to standard guidelines. However, the appropriate regimens were only prescribed 15 months after the initial raised VL.

### Post-intervention

#### Viral load and retention in care review

Post-intervention, 92% (145/158) had VL tests recorded, significantly improving from pre-intervention (*p* = 0.0009) (Table 2). Similarly, filing of results improved post-intervention, with 94% (136/145) of VL results filed. Overall, there was also a significant increase in those who had access to their results (86%; 136/158) (*p* < 0.0001).

#### Viral load management review

Post-intervention, 26% (18/68) were lost to follow-up, 6% (4/68) had transferred out and 1% (1/68) was uncategorised. Twelve per cent (8/68) of patients had a follow-up VL showing lower detection limit (LDL; <400 RNA copies/mL) and 6% (4/68) had VL < 1000 RNA copies/mL negating their need for regimen change, 10% (7/68) of patients already had a regimen change (as per the pre-intervention VL management review above). Of the remaining 26 of 68 patients, 7 (27%) had no regimen changes and 19 (73%) patients had an appropriate regimen change, versus 81% and 11%, respectively, pre-intervention (*p* = 0.0001) (Table 2).

#### Post-intervention clinical summary chart usage review

There were 280 patients newly enrolled on ART at the PHC from 01 April to 30 June 2014. There were 93.6% (262/280) patient files available for assessment.

In this time period, 262/280 files were available: 57% (149/262) of patients assessed were commenced on ART at Lancers Road, while 46% (121/262) were transferred in, already on ART.

A clinical summary chart was present in 85% (224/262) of available patient files (Table 2). Antiretroviral therapy was documented on the clinical summary sheet in the majority (209/224, 93%) of patient files. In 98% (205/209), the ART regimen was up-to-date.

#### Post-intervention down-referral system

Approximately one-quarter of patients were assessed as not applicable either because of being transferred out (5%; 14/262) or lost to follow-up (17%; 44/262), with (78%; 204/262) still in care. Of these, 79% (161/204) would have been eligible for down-referral to ‘chronic clubs’ using the following criteria: patient on ART > 12 months; latest VL result is undetectable/LDL (use of clinical summary chart to see date of latest LDL); not currently pregnant or breastfeeding (needs more frequent monitoring); no recent change in regimen (needs more frequent monitoring) and clinically well. The remainder 21% (43/204) were ineligible for ‘down-referral’ to chronic clubs. The reasons for ineligibility were the following: (1) clinical factors (8%; 17/204) such as pregnancy, raised VL or blips, recent change to new regimen or illness; (2) management error (5%; 10/204) such as VLs not being tested; and (3) missed visits (8%; 16/204).

## Discussion

This project shows that significant improvements in VL testing and monitoring can be achieved through a combination of multifaceted interventions, including the implementation of training, review of patient files and the strategic addition of monitoring systems. We report improvements in the VL tests conducted and the proportion of patients who had access to VL results, which improved by just over 25%. In addition, improvements in management of HIV-positive patients with raised VLs were noted, with a significant reduction in the number of patients who did not require a regimen change, and a significant increase in appropriate regimen changes post-intervention.

In a South African study modelling national and provincial HIV data, 78% of HIV-positive adults on ART had suppressed VLs in 2015; however, only 38% of HIV-positive adults overall had virological suppression.^13^ Better virological suppression rates were reported in KwaZulu-Natal with 85% of HIV-positive patients on ART with undetectable VLs, but only 47% HIV-positive adults were virologically suppressed.^13^ A notable finding of this study was that 73% of the patients who had a VL result available pre-intervention were virologically suppressed, lower than the national (78.4%) and provincial estimates (excluding Limpopo and Mpumulanga).^13^ Virologic suppression was higher in this study than described in other South African ART cohorts.^14^ However, under two-thirds of patients had an available VL test and patients with high VLs were not appropriately managed.

The outcomes measured showed the effectiveness of the interventions, particularly the availability of VL results for clinical management improving from 59% pre-intervention to 87% post-intervention. There was also an improvement in management of raised VLs, with a significant increase in repeat VL tests from 78% pre-intervention to 92% post-intervention. Furthermore, there was a significant increase in appropriate regimen changes from 11% pre-intervention to 73% post-intervention, and timelier follow-up of these patients.

The findings of this study with a quality improvement intervention focusing on the VL monitoring and recognition of treatment failure are similar to other studies in South Africa and Malawi. In a South African study in three clinics in eThekwini in KwaZulu-Natal, a VL champion was designated to focus on virologic management; following the intervention, the VL tests performed significantly increased from under 70% at the three clinics to over 80%.^15^ In a study at 13 facilities in Malawi over a 6-month period, changing staff roles with a VL ‘focal person’ to oversee all VL activities improved VL testing 164% pre-intervention versus post-intervention.^16^

Furthermore, patients are vulnerable to non-adherence and disengagement the longer they remain on treatment.^17^ In a retrospective cohort study in Cape Town, South Africa, almost 23% of patients disengaged from care within 2 years of ART initiation with 16% requiring hospital admissions and 3% dying following disengagement.^17^ Moreover, the WHO-defined VL threshold (VL ≥ 1000 copies/mL) for intervention of virological failure may not adequately identify patients at risk for adverse clinical outcomes. In a recent South African study with over 69 000 patients, HIV-positive patients on ART with low-level viraemia (≥ 50 copies/mL) were threefold more likely to develop virologic failure than patients with undetectable VL; the risk increased to almost fivefold when using VL 400 copies/mL – 999 copies/mL.^18^

According to the South African ART guidelines, an elevated VL (> 400 RNA copies/mL) indicates either poor treatment adherence or resistance, necessitating adherence support and drug therapy changes for treatment optimisation.^11,19^ Low adherence may also lead to virological failure, transmission and drug resistance risk.^20,21,22^ South African data highlight the increasing resistance patterns exacerbated by patients with an unsuppressed VL.^23^

There were several contributory factors to the success of our intervention. The project was conducted in a well-functioning public health system with an inculcated culture of reflective data analysis and quality improvement. The NHLS system facilitated the use of data to drive improvement as it is linked to patient data that are meaningful to frontline staff. The health system leadership used VL process data feedback to encourage participation in learning opportunities and supported the testing of new ideas and the spread of successful interventions.

Another factor for success was the perceived added value and acceptance of the intervention in the healthcare setting by the staff who were using it. This acceptance is reflected by the large proportion of patient files with clinic summary sheets inserted by clinic staff. These results are also indicative of the feasibility of using summary sheets in busy healthcare settings. The clinical summary sheet also proved useful for the completion of the required governmental forms for audit purposes. While there were fields to assist with integrated care provision such as pregnancy or HIV-positive patients co-infected with TB, the clinical summary chart was mainly used for ART management, and assessment of patient eligibility for down-referral to a chronic club. While the initial investment in summarising patient data may be unwieldy, long term, the summary sheet may mitigate the necessity to peruse through long and often complex clinical notes, saving time for both the patient and provider during the consultation. Moreover, if there is substantial buy-in from staff, a clinical summary sheet could be inserted for all patients newly initiating on ART or pre-inserted into files of patients presenting for care the next day.

There is limited data on the association between summary sheets and improvement in clinical care; however, the integrated summary of prospectively collected clinical information can assist clinical staff in organising complex patient data to facilitate management.^24^

In South Africa, HIV care is decentralised to PHC level; ART initiation and follow-up care are primarily nurse-managed with task shifting to less specialised health workers. Although there are clear recommendations set out by the South African ART treatment guidelines on the management of HIV-positive patients,^19,25^ there is inadequate time for nurses to provide comprehensive care because of the large volumes of patients. With the focus on dispensing ART, opportunities for detailed clinical assessment, adherence and psychological support, which are crucial for the success of therapy, are missed, creating major challenges for HIV-positive patients presenting with complex disease to be managed timeously and appropriately.

### Limitations

Assessment of long-term effectiveness of interventions implemented during this operational research is difficult to measure accurately for the following reasons: difficulty of measurable end points; the presence of the research clinicians pre- and post-intervention, with availability for assistance and advice, and a split focus between intervention and review; and the progressive nature of the research, with the need to design tools for measurement while interventions are occurring.

The multifaceted interventions in this study included support from two part-time research clinicians with HIV experience and research assistants who were actively involved in patient care. Hence, while the interventions were fairly simple, it may not be feasible to institute this level of health systems strengthening at all clinics. Moreover, it is difficult to assess which intervention had the most effect given the package of care. Finally, while there was some emphasis on empowering patients in their HIV care, the effect of this on knowledge and behaviour was not measured.

Follow-up of patients not returning to the clinic was difficult to assess because there was no appointment system in place, and missed visits were not easily identified until a patient returned to the clinic and the file was taken out, or, as in this study, all the files reviewed. Despite this, retention in care was 88% and 79% in the in-depth review and the VL retention in care review, respectively, possibly indicative of the commitment of patients to the care they were receiving at the Lancers’ Road PHC. In a prior retrospective study of South African ART cohort, overall retention rates fell from 71% at 24 months to 56% at 60 months; notably these study sites were well resourced.^25^ Currently, programmatic data are limited regarding retention in care in adults in South Africa; studies evaluating postpartum retention in care in PMTCT also noted challenges in attrition because of patient-level factors (disclosure to spouse and competing priorities), provider-level factors (clinical knowledge and attitudes) and wider health systems barriers, such as access to care and drug stock-outs.^26^ The attrition rate following access to universal ART is unclear and there may be a larger loss to follow-up following the 2016 guideline revision to initiate all patients on ART, irrespective of CD4^+^ count.^27^

The gaps in patient care at the PHC clinic may be accounted for by the high patient volume, and prioritisation of ART initiation and dispensing without the assistance of a pharmacist. Moreover, there were few opportunities for clinicians to mentor nurses. Clinical mentoring has been highlighted by WHO to be a vital strategy for the scale of ART and improving healthcare in developing countries.^28^

As this study was conducted at a municipality PHC, it may not be generalisable to other PHC facilities in South Africa. However, it may be feasible to implement most of the lessons learned in improving the quality of virologic management in this study.

## Conclusion

In this study, multifaceted interventions focusing on laboratory systems, staff training and mentoring, streamlined documentation and a strong referral and follow-up system contributed to improvements in virologic management in the time period evaluated.

The third ‘90’ UNAIDS 2020 target is therefore achievable by the implementation of simple, appropriate and targeted systems in a busy PHC setting.

### Recommendations

While it may not be feasible to place extra clinicians at clinics for the sole purpose of mentoring and documentation review, several of the interventions are easy to implement in a resource-poor setting. This includes the clinical summary chart as a simple way to monitor VL status. Moreover, routine education of clinical staff on basic South African governmental protocols is achievable. Finally, with the introduction of Tier.net, an electronic database for HIV-positive patients and access to the NHLS laboratory results system, improvements to the result availability are feasible.
